# Active surveillance versus immediate surgery in the management of low-risk papillary thyroid microcarcinoma: comparison of long-term costs in Brazil

**DOI:** 10.20945/2359-4292-2023-0349

**Published:** 2024-07-12

**Authors:** Fernanda Nascimento Faro, Antônio Augusto Tupinambá Bertelli, Nilza Maria Scalissi, Adriano Namo Cury, Rosália do Prado Padovani, Carolina Ferraz

**Affiliations:** 1 Divisão de Endocrinologia Departamento de Medicina Irmandade da Santa Casa de Misericórdia de São Paulo São Paulo SP Brasil Unidade de Doenças da Tireoide, Divisão de Endocrinologia, Departamento de Medicina, Irmandade da Santa Casa de Misericórdia de São Paulo, São Paulo, SP, Brasil; 2 Serviço de Medicina Nuclear Irmandade da Santa Casa de Misericórdia de São Paulo São Paulo SP Brasil Serviço de Medicina Nuclear, Irmandade da Santa Casa de Misericórdia de São Paulo, São Paulo, SP, Brasil

**Keywords:** Papillary thyroid carcinoma, active surveillance, surgery, cost analysis

## Abstract

**Objective:**

To compare the long-term medical costs of active surveillance (AS), partial thyroidectomy (PT), and total thyroidectomy (TT) in patients with low-risk papillary thyroid microcarcinoma (PTMC) receiving care covered by the Brazilian Public Health System.

**Materials and methods:**

After reviewing AS cohorts and our own data, we created a model of AS, PT, and TT flow care for low-risk PTMC over 10, 20, and 30 years. The medical costs included those associated with diagnosis, surgery, and follow-up. We considered that 13.3% of the patients on AS would require surgery after a mean of 21.3 months, 4% undergoing TT would develop permanent hypoparathyroidism, and 43% undergoing PT would develop hypothyroidism.

**Results:**

The most economical alternative was AS. The costs of TT per patient were higher than those of AS by 182.8% over 10 years (866.89 versus 306.49 US dollars [USD], respectively), by 152.9% over 20 years (1,023.66 versus 404.73 USD, respectively), and by 134.7% over 30 years (1,180.42 versus 502.96 USD, respectively). The costs of PT per patient were higher than those of AS by 16.0% over 10 years (355.66 versus 306.49 USD, respectively), by 16.9% over 20 years (473.41 versus 404.73 USD, respectively), and by 17.5% over 30 years (591.17 versus 502.96 USD, respectively).

**Conclusion:**

The AS approach was less costly than immediate surgery throughout 30 years of follow-up. Hence, the implementation of AS in Brazil should not be hindered by cost considerations.

## INTRODUCTION

The management of low-risk papillary thyroid microcarcinoma (PTMC) is controversial. The traditional approach – i.e., immediate surgery with total thyroidectomy (TT) – has been considered excessive given the reduced rates of recurrence and mortality associated with the disease and the potential for surgical complications (1-3). According to guidelines by the American Thyroid Association (4), patients with unifocal and intrathyroidal PTMC without clinically detectable cervical lymph node metastasis may be treated with partial thyroidectomy (PT) alone to reduce short-term and long-term postoperative morbidity (5). Additionally, many cases of PTMC may not require surgery, and active surveillance (AS) has been demonstrated to be a safe management strategy in these cases (6,7).

The AS protocol involves monitoring signs of disease progression with periodic ultrasound assessments (8). If disease progression is detected, surgical therapy is recommended. According to several studies on AS, the majority of low-risk PTMCs do not progress, and none of the patients included in these studies developed distant metastasis or died from PTMC because of delayed surgery (6,7,9-15). Still, the economic impact of long-term follow-up in AS has been questioned and remains a barrier to the acceptance of this management approach.

Cost analyses of AS for PTMC have methodological limitations and exhibit variability based on clinical protocols, follow-up duration, and the country where the analysis is conducted (16). Therefore, costs should be considered individually for each specific region. To date, no studies have been conducted comparing the costs of managing low-risk PTMC in Latin America.

Brazil has a robust public health system (*Sistema Único de Saúde* [SUS]) that covers care for all legal citizens. The system offers many services free of charge, including medical consultations, exams, surgeries, hospital services, and medications. However, the wait for some types of treatment (e.g., thyroid surgery) is long, and patients with indolent diseases like PTMC may have to wait for months or years to undergo surgery. In a way, these patients are undergoing AS but without the appropriate follow-up. Considering these facts, a cost analysis of each treatment alternative for low-risk PTMC in Brazil is critical.

Based on these considerations, the aim of this study was to compare the long-term medical costs of AS, PT, and TT in patients with low-risk PTMC receiving care covered by the SUS.

## MATERIALS AND METHODS

The present study was conducted in accordance with the 2022 Consolidated Health Economic Evaluation Reporting Standards (CHEERS) statement (17).

### Study design

From a review of previous cohorts undergoing AS and analysis of our own data (Table 1), we created a model of AS, PT, and TT flow care for low-risk PTMC over 10, 20, and 30 years, following national (18,19) and international guidelines (4,8) and the clinical practices in our service.

**Table 1:** Clinical studies of active surveillance in patients with low-risk papillary thyroid microcarcinoma

First Author, Country, Year	Number of Patients	Duration of Follow-up in Months (mean)	Conversion from Active Surveillance to Surgery (%)	Duration of Follow-up from Diagnosis to Surgery (months)
Ito, Japan, 2014 (9)	1235	60.0	16.0	-
Tuttle, USA, 2017 (10)	291	25.0	3.5	-
Sanabria, Colombia, 2018 (11)	102	13.9	12.7	12.0
Oh, South Korea, 2018 (12)	370	32.5	15.6	24.8
Rosario, Brazil, 2019 (13)	77	-	4.3	30.0
Smulever, Argentina, 2020 (14)	41	37.5	29.0	-
Molinaro, Italy, 2020 (15)	93	19.0	23.0	8.0
Sugitani, Japan, 2023 (6)	571	91.0	9.5	-
Faro, Brazil, 2023*	23	35.2	9.0	30.0
**Weighted Mean**		55.4	**13.3**	**21.3**

*Unpublished.

Based on data from 2,803 patients with low-risk PTMC on AS (6,9-15), we calculated that 13.3% required surgery during follow-up and that TT was the surgery of choice in these cases. The mean time to conversion from AS to surgery after diagnosis was 21.3 months. Based on our review of the literature (20), we considered a 4% rate of permanent hypoparathyroidism in patients undergoing TT and a 43% rate of hypothyroidism in those undergoing PT (3).

### Follow-up protocols

**Figure 1:**
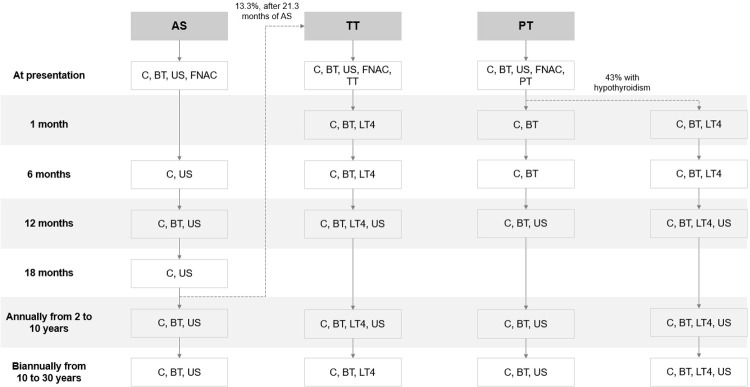
Flowchart of the sequence of procedures in protocols for the management of papillary thyroid microcarcinoma in patients undergoing active surveillance, partial thyroidectomy, and total thyroidectomy.

Abbreviations: AS, active surveillance; BT, blood tests; C, consultation; FNAC, fine-needle aspiration cytology; LT4, levothyroxine; US, ultrasound examination; PT, partial thyroidectomy; TT, total thyroidectomy. Biannually indicates every 2 years. For improved visualization, the flowchart does not include the information that 4% of the patients in the TT group were considered to have permanent hypoparathyroidism and, for these patients, total daily doses of 1,000 mg of calcium carbonate and 0.25 μg of calcitriol were added to the costs.

The flowchart in Figure 1 shows the sequence of procedures in standard protocols for the management of low-risk PTMC. After diagnosis, patients on AS are monitored with medical consultations and ultrasound examinations every 6 months for 2 years, then annually between 3-10 years, and every 2 years between 11-30 years after diagnosis. Patients undergoing surgery immediately after diagnosis are monitored with medical consultations and blood tests at 1 and 6 months and ultrasound examination at 1 year after surgery. After that, ultrasound examinations continue yearly until 10 years after diagnosis for patients who undergo TT, while for those undergoing PT, the follow-up procedures are the same as those for AS (4,19).

### Medical costs

The medical costs considered in the analysis included those associated with the initial diagnosis, surgery, and follow-up care (Figure 1 and Table 2). The values were obtained from cost tables published by the SUS (21) and are shown in Brazilian reais (BRL) and US dollars (USD), considering a conversion rate of 4.90 BRL to 1 USD. The costs associated with both types of surgeries (PT and TT) are based on typical practices at our service and include those related to preoperative assessments, surgery, anesthesia, pathological examination, and hospitalization.

**Table 2:** Costs of major clinical activities, tests, and surgical treatment covered by the Brazilian Public Health System (SUS)

Item	Monthly cost in Brazilian reais	Monthly cost in US dollars
Medical consultation	10.00	2.04
Blood tests*	53.27	10.87
Ultrasound examination	24.20	4.93
Fine-needle aspiration cytology	47.93	9.78
Total thyroidectomy	2877.08	587.15
Partial thyroidectomy	466.41	95.18
Levothyroxine	2.70	0.55
Calcium and calcitriol tablets	26.46	5.4

*Blood tests included measurement of serum TSH, free thyroxine, thyroglobulin, and antithyroglobulin antibody levels. The costs of both surgeries (partial and total thyroidectomy) included general anesthesia, surgical procedures, pathological examinations, and hospitalization fees during the perioperative period. The daily doses of the medications are as follows: levothyroxine, 100 μg; calcium carbonate, 1,000 mg; calcitriol, 0.25 μg.

The blood tests included measurement of serum levels of thyroid-stimulating hormone (TSH), free thyroxine (fT4), thyroglobulin, and antithyroglobulin antibodies. The costs of measuring calcium levels were not included. For patients with postoperative hypothyroidism, we considered a daily dose of levothyroxine of 100 μg. For those with postoperative hypoparathyroidism, we considered daily doses of calcium carbonate and calcitriol of 1,000 mg and 0.25 μg, respectively. The costs of the medications were calculated as those of public bidding for a low-cost generic drug in the last 2 years. The costs associated with lymph node dissection and radioactive iodine (RAI) were not included in the analysis.

### Data analysis

The data were calculated using Microsoft Excel 365. We considered the rates of conversion from AS to surgery described in Table 1 and the mean costs described in Table 3.

**Table 3:** Medical costs associated with active surveillance, partial thyroidectomy, and total thyroidectomy for management of low-risk papillary thyroid microcarcinoma over 10, 20, and 30 years of follow-up

Management time point	Medical costs (mean)*		
	Active surveillance	Partial thyroidectomy	Total thyroidectomy
Diagnosis	27.63 (135.40)	122.81 (601.81)	614.79 (3012.48)
10 years	306.49 (1501.81)	355.66 (1742.71)	866.89 (4247.74)
20 years	404.73 (1983.16)	473.41 (2319.73)	1023.66 (5015.91)
30 years	502.96 (2464.51)	591.17 (2896.74)	1180.42 (5784.08)

*The values are shown in US dollars (Brazilian reais)

We performed a sensitivity analysis to evaluate the impact of the costs associated with procedures and surgical complications on the results and to identify the main cost drivers. In this analysis, we considered cost decreases and increases of 2%, 5%, 10%, 15%, and 20% for each item individually while maintaining the costs of the other items fixed, and we ultimately chose to present the results of a 10% variation from the original cost. We also performed sensitivity analysis while maintaining the costs of each item and adjusting for different conversion rates from AS to surgery.

## RESULTS

**Figure 2:**
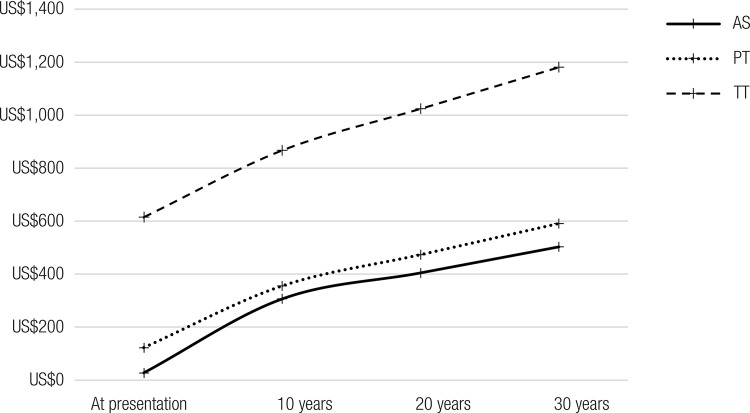
Medical costs associated with active surveillance, partial thyroidectomy, and total thyroidectomy for follow-up and care of patients with low-risk papillary thyroid microcarcinoma over 10, 20, and 30 years.

Abbreviations: AS, active surveillance; PT, partial thyroidectomy; TT, total thyroidectomy.

The results of the analyses are shown in Table 3 and Figure 2. Overall, the total costs were lower for AS than PT or TT.

The costs of TT were 182.8%, 152.9%, and 134.7% higher than those of AS at 10 years, 20 years, and 30 years, respectively. Specifically, the estimated costs per patient managed with TT versus AS were, respectively, 866.89 USD (4,247.74 BRL) versus 306.49 USD (1,501.81 BRL) over 10 years, 1,023.66 USD (5,015.91 BRL) versus 404.73 USD (1,983.16 BRL) over 20 years, and 1,180.42 USD (5,784.08 BRL) versus 502.96 USD (2,464.51 BRL) over 30 years.

The costs of PT were 16.0%, 16.9%, and 17.5% higher than those of AS at 10 years, 20 years, and 30 years, respectively. Specifically, the estimated costs per patient managed with PT versus AS were, respectively 355.66 USD (1,742.71 BRL) versus 306.49 USD (1,501.81 BRL) over 10 years, 473.41 USD (2,319.73 BRL) versus 404.73 USD (1,983.16 BRL) over 20 years, and 591.17 USD (2,896.74 BRL) versus 502.96 USD (2,464.51 BRL) over 30 years.

The differences in costs between TT and AS tended to reduce over the years, while those between PT and AS tended to increase, favoring AS.

When the conversion from AS to surgery was not considered in the analysis, the cost of TT was 164.4%, 133.7%, and 115.3% higher than those for “simple cost” AS. Specifically, the costs per patient for TT versus AS considering this scenario were, respectively, 866.89 USD (4,247.74 BRL) versus 327.86 USD (1,606.51 BRL) over 10 years, 1,023.66 USD (5,015.91 BRL) versus 437.97 USD (2,146.03 BRL) over 20 years, and 1,180.42 USD (5,784.08 BRL) versus 548.07 USD (2,685.54 BRL) over 30 years. The corresponding costs of PT were 8.5%, 8.1%, and 7.8% higher than those for “simple cost” AS, specifically, 355.66 USD (1,742.71 BRL) versus 327.86 USD (1,606.51 BRL) over 10 years, 473.41 USD (2,319.73 BRL) versus 437.97 USD (2,146.03 BRL) over 20 years, and 591.17 USD (2,896.74 BRL) versus 548.07 USD (2,685.54 BRL) over 30 years.

**Figure 3:**
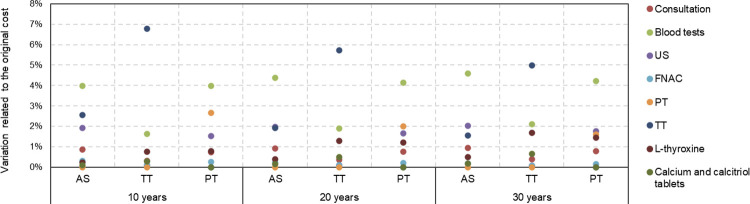
Variation in costs with active surveillance, partial thyroidectomy, and total thyroidectomy over 10, 20, and 30 years of care for patients with low-risk papillary thyroid microcarcinoma considering an increase of 10% for each medical cost individually.

Abbreviations: C, consultation; FNAC, fine-needle aspiration cytology; L-thyroxine, levothyroxine; PT, partial thyroidectomy; TT, total thyroidectomy; US, ultrasound examination.

Figure 3 shows the results of sensitivity analysis evaluating the impact of a 10% variation in the original costs. The items with the greatest impact on costs were blood tests for AS and PT and surgical expenses for TT.

We further estimated the variations in each item cost required to equalize the costs of surgeries and AS at 30 years (Table 4). We found that a 231% increase in the total costs of blood tests would result in equal costs between TT and AS at this time point. In contrast, a 93% reduction in PT costs would be necessary to align the costs of AS and PT. Another sensitivity analysis was conducted maintaining the items’ costs while adjusting the rates of conversion from AS to surgery (Table 5). For the costs of AS to be equal to those of TT and PT at 30 years, the rates of conversion from AS to TT and AS to PT would have to be 24.3% and 52.8%, respectively. If the conversion rates increased above these values, AS would have higher costs than TT or PT.

**Table 4:** Estimated variation in each item cost to equalize the costs of total thyroidectomy and partial thyroidectomy to those of active surveillance for the management of low-risk papillary thyroid microcarcinoma over 30 years of follow-up

	Total Thyroidectomy	Partial Thyroidectomy
Consultation	1,050%	994%
Blood tests	231%	405%
Ultrasound examination	296%	554%
Fine-needle aspiration cytology	5,142%	5,142%
Partial thyroidectomy	-	-93%
Total thyroidectomy	*	96%
Levothyroxine	*	*
Calcium and calcitriol tablets	*	771%

*Scenarios in which the costs should be reduced by more than 100%.

**Table 5:** Analyses maintaining the items’ costs and adjusting the rates of conversion from active surveillance to surgery

		Rates of conversion									
		Original	3.5%	4.3%	9.0%	9.5%	12.7%	15.6%	16%	23%	29%
10 years	Active surveillance	-	-	-	-	-	-	-	-	-	-
	Total thyroidectomy	**2.83**	3.57	3.50	3.11	3.08	2.86	2.70	2.68	2.35	2.12
	Partial thyroidectomy	**1.16**	1.46	1.43	1.28	1.26	1.18	1.11	1.10	0.96	0.87
20 years	Active surveillance	-	-	-	-	-	-	-	-	-	-
	Total thyroidectomy	**2.53**	3.06	3.01	2.74	2.71	2.56	2.43	2.41	2.16	1.98
	Partial thyroidectomy	**1.17**	1.42	1.39	1.27	1.25	1.18	1.12	1.12	1.00	0.92
30 years	Active surveillance	-	-	-	-	-	-	-	-	-	-
	Total thyroidectomy	**2.35**	2.77	2.73	2.52	2.49	2.37	2.27	2.25	2.04	1.89
	Partial thyroidectomy	**1.18**	1.39	1.37	1.26	1.25	1.19	1.13	1.13	1.02	0.94

## DISCUSSION

Papillary thyroid carcinoma (PTC), the most common type of thyroid carcinoma, has nearly tripled in incidence in the last decades. This rise has been mainly due to increased detection of low-risk PTMCs (i.e., PTCs ≤ 1 cm) rather than clinically significant PTCs, as the increasing incidence does not appear to have affected the morbidity or mortality rates of the disease (22,23). This finding suggests a potential issue of overdiagnosis, overtreatment, and likely avoidable morbidity related to the treatment of this disease, prompting the hypothesis that low-risk PTMCs may not warrant immediate surgery.

Results from a clinical trial of AS for low-risk PTC conducted at Kuma Hospital in Japan over 30 years (1993-2023) showed excellent and similar outcomes for AS and immediate surgery (7). This finding has been confirmed in several other trials of AS for low-risk PMC conducted in different countries (6,7,9-15). An important point to consider is that surgical treatment may be associated with unfavorable events like hypothyroidism, iatrogenic hypoparathyroidism (7%-37% of the cases), and permanent recurrent laryngeal nerve palsy (0.9%-5.9%) (24-26).

While the safety of adopting AS in low-risk PTMC has been demonstrated, concerns about its feasibility and perceived economic impact have raised questions regarding its widespread acceptance. Differences in medical care systems and costs across countries are substantial. Therefore, cost analyses of AS compared with immediate surgery should be conducted considering each country’s medical care system (16). For this reason, we conducted the present analysis to evaluate the economic feasibility of AS considering the medical costs of the SUS.

This is the first study comparing long-term medical costs associated with AS, PT, and TT for low-risk PTMC in Latin America. The results indicate that the total costs of AS were lower than those for TT or PT at diagnosis and continued to be lower throughout 30 years of follow-up. These results are aligned with findings from similar studies in the literature. A systematic review analyzing the cost-effectiveness of AS compared with immediate surgery found that most studies favored AS (27). Oda and cols. (28) reported that the total costs of immediate surgery with postoperative care for 10 years were 4.1 times higher than those associated with AS at the Kuma Hospital in Japan. In contrast, Lin and cols. (29) found that the estimated cost of surgical PTMC treatment was equivalent to the cost of AS lasting 16.2 years in Australia. However, these authors considered that patients undergoing hemithyroidectomy were examined with ultrasound for 5 years and considered cured after that. According to guidelines from the American Thyroid Association (4), the ideal frequency of follow-up assessments in patients who do not undergo RAI ablation is uncertain. A position statement from the Thyroid Department of the Brazilian Society of Endocrinology and Metabolism on treatment strategies for low-risk PTMC emphasizes that the follow-up of patients treated with lobectomy should be based mainly on results from neck ultrasound, that tumor recurrence occurs in about 5% of all patients, and that the emergence of new nodules is not infrequent (19). Based on these facts, we considered in our study that the clinical follow-up after PT should be the same as that suggested for AS.

Lin and cols. (29) applied sensitivity analysis to assess the cost efficiency of decreasing the follow-up interval for AS from twice to once a year and found that this change markedly reduced the cost of AS, surpassing that of surgical treatment after 45.1 years. The Consensus Statements from the Japan Association of Endocrine Surgery Task Force on Management for Papillary Thyroid Microcarcinoma (8) recommend ultrasound evaluations every 6 months for 1-2 years after initiation of AS and, if no disease progression is detected, once every 1 or 2 years after that. Tuttle and cols. (30) analyzed the kinetic patterns of tumor growth in patients with low-risk PTC and found that these tumors grow at a slow rate and double in volume at 2-3 years, with most tumors reaching a stable volume after 5 years. In our review of AS cohorts, we found that the mean time for conversion from AS to surgery was 21.3 months after diagnosis. Thus, our protocol includes ultrasound twice a year in the first 2 years, then yearly until 10 years, and every 2 years after that.

To analyze the “simple cost” of AS while taking into consideration that anxiety is a major cause of conversion from AS to surgery, we performed a subanalysis excluding conversion surgery and found that the difference between the “simple cost” of AS and immediate surgery became even more expressive. This finding has also been reported by Oda and cols. (28). In this case, the cost difference between AS and PT reduces over time, but PT remains more expensive than AS after 30 years.

A limitation of our study is the lack of cost-effectiveness analysis. Lang and cols. (31) compared the estimated cost-effectiveness between AS and immediate surgery in terms of quality-adjusted life-years (QALYs) gained. In their study, each patient who chose AS over immediate surgery cost an extra 682.54 USD but gained an additional 0.260 QALY. The authors concluded that AS was less costly and more effective up to 16 years from PTMC diagnosis and remained cost-effective from 17 years onward. Applying sensitivity analysis, they found that AS remained cost-effective regardless of patient age (<40 or ≥40 years), complications, progression rates, year cycle, or discount rate.

Our study has some other limitations. First, we did not perform a meta-analysis to calculate the percentage of patients converting from AS to surgery during follow-up. A meta-analysis by Issa and cols. found that 12% of patients on AS undergo surgery, which is very close to the percentage of 13.3% found in our study (32). However, we included a calculation of the total costs using different conversion rates and demonstrated that a higher conversion rate from AS to surgery would be necessary for the costs of AS to be equal to those of TT and PT. Second, we did not evaluate the impact of patients’ age at diagnosis on costs. Using the Kaplan-Meier method, Miyauchi and cols. (2) estimated the disease progression rate at the 10-year time point for AS at each age group divided into decades (20s to 70s) and found a lifetime risk of disease progression over 60% in patients aged 20-30 years. Based on that, younger patients would be less cost-efficient in an AS program. Third, our study underestimated costs, as we derived the costs from the Table of Procedures, Medications, and Orthoses, Prostheses, and Special Materials (SIGTAP) (21); this SUS instrument, created and updated by the government, publishes reference values paid to professionals and health care institutions. However, there is a gap between the SIGTAP values and the actual amounts paid, which is why the surgical procedures costs were underestimated, and the PT cost at presentation was so close to the AS cost. In our analysis, the isolated cost of surgery was the variable with the greatest impact on the total cost of TT. Additionally, the costs of the SUS are very different from those of private health insurance and services. These services were not considered because their values are not standardized and vary greatly, hindering a proper comparison. Also, we did not include the costs of lymph node dissection, RAI, and those associated with vocal cord palsy. However, we believe that, with the inclusion of these factors, the surgery cost would be slightly higher, which would favor AS. Discount rates were not considered because, in our opinion, they would be similar for all the procedures and would not influence the analysis result.

In conclusion, cost analyses have several methodological limitations, and costs may differ due to clinical heterogeneity and protocols, follow-up duration, medical costs specific to each country, public versus private systems, and criteria used in the analysis (e.g., age and quality of life). Our comparison of costs of AS versus immediate surgery (PT and TT) in managing low-risk PTMC showed that AS is the least costly strategy throughout 30 years of follow-up care. Thus, the economic impact on public health care should not be a barrier to implementing AS for patients with low-risk PTMC in Brazil.
